# Validation of QTLs associated with corn borer resistance and grain yield: implications in maize breeding

**DOI:** 10.3389/fpls.2024.1404881

**Published:** 2024-10-22

**Authors:** Ana López-Malvar, Zoila Reséndiz-Ramirez, Ana Butrón, Jose Cruz Jiménez-Galindo, Pedro Revilla, Rosa Ana Malvar

**Affiliations:** ^1^ Facultad, de Biología, Departamento de Biología Vegetal Y Ciencias del Suelo, Universidad de Vigo, Vigo, Spain; ^2^ Agrobiología Ambiental, Calidad de Suelos Y Plantas (UVIGO), Unidad Asociada a La MBG (CSIC), Vigo, Spain; ^3^ Instituto Nacional de Investigaciones Forestales, Agrícolas y Pecuarios, Río Bravo, Tamaulipas, Mexico; ^4^ Misión Biológica de Galicia (CSIC), Pontevedra, Spain; ^5^ National Institute of Forestry Agriculture and Livestock Research (INIFAP), Cuauhtémoc, Chihuahua, Mexico

**Keywords:** Mediterranean corn borer, QTL validation, maize breeding, MAGIC population, grain yield, recombinant inbred lines

## Abstract

**Introduction:**

Validations of previously detected quantitative trait loci (QTLs) to assess their reliability are crucial before implementing breeding programs. The objective of this study was to determine the reliability and practical usefulness of previously reported QTLs for resistance to stem tunneling by the Mediterranean stem borer (MSB) and yield. These authors used approximately 600 recombinant inbred lines (RILs) from a multiparent advanced generation intercross (MAGIC) population to map QTL using a genome-wide association study (GWAS) approach.

**Methods:**

We identified RILs situated at the extremes of resistance and yield distributions within the whole MAGIC, and those QTLs were evaluated *per se* and crossed to a tester (A638) using lattice designs. In each set, a significant single-nucleotide polymorphism (SNP) was considered validated if (1) the same SNP was associated with the trait with a *p*-value < 0.02, or (2) within a ±2-Mbp interval, an SNP associated with the trait exhibited a *p*-value < 0.02 and demonstrated linkage disequilibrium (*r*2 > 0.2) with the SNPs previously reported.

**Results and discussion:**

The novel QTL validation approach was implemented using improved experimental designs that led to higher heritability estimates for both traits compared to those estimated with the whole MAGIC population. The procedure used allowed us to jointly validate several QTL and to ascertain their possible contribution to hybrid improvement. Specifically, nearly three-quarters of the QTLs for tunnel length were confirmed. Notably, QTLs located in the genomic region 6.05–6.07 were consistently validated across different sets and have been previously linked to resistance against stalk tunneling in various mapping populations. For grain yield, approximately 10 out of 16 QTLs were validated. The validation rate for yield was lower than for tunnel length, likely due to the influence of dominance and/or epistatic effects. Overall, 9 out of 21 QTLs for tunnel length and 6 out of 17 QTLs for grain yield identified in our previous research were validated across both validation sets, indicating a moderate genetic correlation between *per se* and testcross performance of inbred lines. These findings offer insights into the reliability of QTL and genomic predictions, both derived from assessments conducted on the entire MAGIC population. Genomic predictions for tunnel length based on inbred line evaluations could be useful to develop more resistant hybrids; meanwhile, genomic prediction for yield could only be valid in a homozygous background.

## Introduction

The primary pest affecting maize in Europe is the Mediterranean corn borer (MCB) *Sesamia nonagrioides* (Lef.). Stem corn borer larvae feed on the stem pith of maize producing tunnels that cause yield losses of approximately 30%, equivalent to worldwide losses of 311.3 million tons every year (Meissle et al., 2010). Tunnels produced in the stalk pith interfere with assimilate movement of nutrients toward the developing ear and increase lodging rate (López et al., 2001). One approach to pest control involves the cultivation of resistant and/or tolerant varieties, particularly in regions where the cultivation of transgenic maize is prohibited or heavily regulated, such as in Europe (Farinós et al., 2004). Genetic improvement of pest resistance relies on attaining varieties that experience reduced damage produced by insects. However, the most resistant varieties could not be the most productive in terms of grain yield. Even more, certain materials have shown a negative correlation between resistance to stem borer attack and grain yield (Sandoya et al., 2008; Butrón et al., 2012). In those cases, selection of varieties that are able to produce higher grain yields under high insect pressure could be a better alternative (Butrón et al., 1999; Ordas et al., 2012). However, at a particular site, the pest infestation level is influenced by multiple factors, exhibiting erratic behavior across years, and differences among varieties for grain yield could be highly affected by the infestation rate. Therefore, it is important to assess varieties’ performance across different scenarios concerning insect attack intensity in order to select varieties with high stable yield.

In order to optimize the development of varieties with reduced damage by insect attack and/or high yield across different scenarios, it is imperative to comprehend the inheritance of the traits involved and to study the regions of the genome or quantitative trait loci (QTLs) associated with resistance using different mapping populations in order to advance in the complete QTLome knowledge for these highly relevant traits (Salvi and Tuberosa, 2015). Accordingly, we can devise the optimal selection method (whether genomic or phenotypic), determine the feasibility of managing any QTL through marker-assisted selection (MAS), or even investigate genes involved in the inheritance of insect resistance. In the specific case of resistance to corn borers, QTLs have been widely sought using biparental populations and linkage-based QTL mapping (Ordas et al., 2009; Samayoa et al., 2014; Samayoa et al., 2015a, 2017; Jiménez-Galindo et al., 2017). However, the use of high-density molecular markers is now widespread due to its low cost, and genome-wide association studies (GWAS) are being conducted for a wide range of traits, including maize resistance to insects (Butrón et al., 2018). GWAS for resistance to the MCB have been conducted using diversity panels of inbred lines (Samayoa et al., 2015b) and multiparent advanced generation intercross (MAGIC) populations (Jiménez-Galindo et al., 2019).

Although many QTLs for resistance to stem corn borers have been found, and molecular markers significantly associated to resistance have been identified, MAS (Flint-Garcia et al., 2003; Samayoa et al., 2019) and genomic selection (Gesteiro et al., 2023) have been scarcely used to improve maize resistance to insects. External validations of detected QTL to assess QTL reliability are advisable before implementing MAS. QTL reliability can be affected by a significant QTL × environment interaction but also by low phenotyping precision. 

Phenotyping genetically wide panels of inbred lines or RILs is challenging due to the difficulty of controlling the high experimental error associated with large trials; mostly when evaluations are often done using incomplete block or augmented designs in which most genotypes are assessed only once in each trial. Moreover, assessing plant traits related to performance against pest attack involves managing pest populations that could show variability for survival, voracity, or any other trait that would affect plant performance. Therefore, the high experimental errors associated with these evaluations could greatly interfere with QTL identification.

In addition, reliable additive-effect QTL could not be useful to discriminate among heterozygous genotypes if heterotic effects can counterbalance additive effects. Therefore, QTL validation should be done using inbred lines as well as hybrids to really ascertain the practical value of detected QTLs. Hence, the objective of this study was to determine the reliability and practical usefulness of QTL for resistance and yield reported by Jiménez-Galindo et al. (2019). Identification of QTL was based on a GWAS approach applied to a maize MAGIC population evaluated using augmented designs in a 2-year evaluation. For validation purposes, we selected inbred line sets at the tails of distributions for resistance and yield in the whole MAGIC to maintain genetic variability for both traits. These inbreds were evaluated *per se* and crossed to a tester (A638) using lattice designs. The results obtained in this study will contribute to expanding the maize QTLome knowledge for traits highly relevant for maize breeding as the they are related to pest tolerance and resistance.

## Materials and methods

### Field experiments

The Maize Genetics and Breeding group of the Misión Biológica de Galicia has developed an eight-way MAGIC population of 608 RILs. This MAGIC population was evaluated for resistance to corn borer attack and grain yield for 2 years (2014 and 2015) using augmented designs (Jiménez-Galindo et al., 2019). Data compiled by these authors are provided in a **Supplementary Table**.

Based on those evaluations, we selected a group of lines presenting either the longest or shortest tunnels made by MCB attack and/or exhibiting the highest or lowest yield. A collection of 56 RILs [inbred line validation set (IVS)], representing the widest variability for these two traits, was then established as the basis for this study. This set of RILs was crossed with A638 to obtain hybrids to validate QTL in a heterozygous background [hybrid validation set (HVS)]. The tester was chosen to avoid interferences on differences among hybrids due to divergences in heterosis as the inbred A638 belongs to the “Reid” group while RILs are characterized by the lack of Reid material in their pedigrees.

The IVS was evaluated along with the eight inbred checks used in previous evaluations (Jiménez-Galindo et al., 2019). Five of the checks were founders of the MAGIC population (A509, EP125, EP17, EP86, and F473), which are partially resistant to corn borer attack. The other three inbred checks were susceptible inbreds, EP42, EP47, and EP80 (Butrón et al., 2006). The 64 lines were evaluated in Pontevedra, in 2017 and 2018, following simple 8 × 8 lattice designs in two adjacent trials, one treated with insecticide (INSECT 5G, Chlorpyrifos 5%) (Control) and the other under artificial infestation with *Sesamia nonagrioides* eggs. The first insecticide treatment was applied between the sixth and seventh week after sowing, and treatment was repeated every 3 weeks until harvest. 

Before flowering, in the trial under artificial infestation, pest infestation was ensured by placing ~80–120 MCB eggs between the sheath and the stem in one internode below the main ear on five plants per plot. Eggs were obtained from insect rearing in the MBG-CSIC laboratory following the methodology of Eizaguirre and Albajes (1992). In all inbred trials, the elementary plot consisted of 15 plants sown at a density of 70,000 plants/ha.

Fifty-two hybrids (HVS), obtained by crossing 52 out of the 56 selected inbreds with A638, along with four hybrid checks were also evaluated. Evaluations were made in 2017 and 2018 in Pontevedra under insecticide treatment and infestation with MCB eggs, in trials adjacent to those described for inbreds, and in Barrantes (42°29′N; 8°45′O, 115 m above sea level) and Ponte Caldelas (42°23′N 8°30′O, 291 m above sea level) in 2017 under natural infestation conditions. Natural infestation was high in both locations. The experimental design employed was a simple 8 × 7 lattice, with each elementary plot consisting of 25 plants and a density of 70,000 plants per hectare.

At harvest, grain yield per plant and plot grain yield were determined and expressed in g plant^−^1 and Mg ha^−^1 at 14% grain moisture content in IVS and HVS, respectively. In the artificial infestation trials, the five infested plants per plot were collected; the stalks were split lengthwise, and the lengths of the tunnels made by the larvae were measured. A similar procedure was used to measure damage by larvae on naturally infested hybrid trials, although it was done on five random plants per plot.

### Statistical analysis

#### Inbred lines

A combined analysis of variance across years and condition (control and infested) was carried out for grain yield and across years at infestation conditions for tunnel length. The inbred lines were considered as fixed factors while the rest of the main factors and interactions were considered as random factors. Comparison of means was done using Fisher’s protected LSD. 

The best linear unbiased estimators (BLUEs) across environments were calculated for each RIL using the Proc Mixed procedure of SAS, with genotype being considered as a fixed factor and year and block as random factors. Heritabilities on a family mean basis were calculated following Holland et al. (2003). To estimate genotypic, residual, and genotype × environment variance, we implemented the VARCOMP procedure in SAS considering all factors as random. The phenotypic variance was calculated as the sum of genotypic, residual, and genotype × environment variance.

#### Hybrids

A combined analysis across years and conditions was performed for grain yield and tunnel length (excluding protected trial data). Hybrid means were compared using Fisher’s protected LSD and BLUEs across environments were estimated for each RIL. Heritabilities and variance components were computed as explained above. All analyses were carried out with SAS software (SAS/STAT, 2007).

### Association mapping

GWAS, based on a mixed linear model that includes a genotype–phenotype matrix, was completed with Tassel 5 (Bradbury et al., 2007) on inbred lines and hybrids, separately. Restricted maximum likelihood estimates of variance components were obtained using the “compressed MLM” and “previously determined population parameters” (P3D) methods described by Zhang et al. (2010). The BLUEs comprised the phenotypic matrices while the genotypic matrix consisted of 224,363 single-nucleotide polymorphisms (SNPs), the same matrix employed by Jiménez-Galindo et al. (2019).

In order to validate the SNPs described as significantly associated with grain yield and tunnel length by Jiménez-Galindo et al. (2019), in the current study, the linkage disequilibrium of significant (*p-*value < 0.02) marker–phenotype associations with published significant SNPs was studied. In the respective set (inbreds and hybrids), a published significant SNP (from Jiménez-Galindo et al., 2019) was considered validated if (1) the same SNP was associated with the trait with a *p-*value < 0.02, or (2) within a ±2-Mbp interval, there were SNPs associated with the trait at *p*-value < 0.02 and in linkage disequilibrium (*r*2 > 0.2) with the SNPs reported by Jiménez-Galindo et al. (2019).

## Results

### Means, heritability, and variance components

We observed significant differences among inbred checks and among RILs in IVS and HVS (data not shown). BLUE estimates for inbreds and hybrids are shown in **Supplementary Tables 2** and **3**, respectively. Regarding heritability estimates, we observed an increase in the values obtained using the set of lines showing extreme values for both traits compared to the values obtained using the whole MAGIC population (Jiménez-Galindo et al., 2019) (**Table 1**). We studied the decomposition of phenotypic variance in tunnel length and grain yield. Genotype × environment interaction variance is minimal in both traits, in both magnitude and proportion, though significant in IVS. In tunnel length, genetic variance prevails in IVS, less so in HVS and the entire MAGIC population. In grain yield, genetic variance remains consistent in IVS and HVS but decreases in the whole MAGIC population.

**Table 1:** Heritability, means, range, and variance components calculated with the whole MAGIC population, the inbred validation set (IVS), and the hybrid validation set (HVS).

	Tunnel length				Grain yield			
Heritability (h2) ^1^								
Whole MAGIC	0.24 ± 0.08*				0.46 ± 0.04*			
IVS	0.74 ± 0.08				0.67 ± 0.08			
HVS	0.52± 0.11				0.74 ± 0.06			
Means								
Whole MAGIC	35 cm				42.4 g/plant			
IVS	25 cm				73.3 g/plant			
HSV	29 cm				7.6 t/ha			
Range								
Whole MAGIC	0–59 cm				0–114 g/plant			
IVS	6–63 cm				20–117 g/plant			
HSV	16–40 cm				4.6–9.4 t/plant			
Variances								
	**Vg2**	**Vgxe3**	**Vres4**	**Vp5**	**Vg**	**Vgxe**	**Vres**	**Vp**
Whole MAGIC	21 ± 4	7 ± 5	91 ± 5	119	78 ± 10	3 ± 6	186 ± 9	267
IVS	107 ± 30	38 ± 17	75 ± 12	220	229 ± 70	174 ± 65	582 ± 61	985
HVS	21 ± 9	17 ± 12	119 ± 13	157	118 ± 33	40 ± 31	396 ± 37	555
	**Vg/Vp**	**Vgxe/Vp**	**Vres/Vp**		**Vg/Vp**	**Vgxe/Vp**	**Vres/Vp**	
Whole MAGIC	0.23	0.08	0.76		0.29	0.01	0.70	
IVS	0.49	0.17	0.34		0.23	0.18	0.59	
HVS	0.14	0.11	0.75		0.21	0.07	0.71	

^1^Heritability estimated on family basis ± standard error; 2 Genotypic variance; 3 Genotype × environment variance; 4 Residual variance; 5 Phenotypic variance. *A variance is significant when it exceeds twice the standard error.

### Validation of QTL for tunnel length produced by *Sesamia nonagrioides larvae*

None of the SNPs that showed significant associations with tunnel length in the whole MAGIC (Jiménez-Galindo et al., 2019) showed significant associations (*p* < 0.02) with tunnel length in the IVS and only one in the HVS. However, within both sets, we have pinpointed SNPs that show a significant association with tunnel length falling within the confidence intervals of SNPs significantly linked to the trait across the entire MAGIC population.

Using the IVS, all of the QTLs detected with the whole MAGIC on chromosomes 5, 6, and 7 were validated. In addition, half of the QTLs on chromosomes 1, 2, and 8 were validated along with one out of three QTLs on chromosome 3. None of the six QTLs on chromosome 4 has been validated in IVS.

Moreover, using HVS, the same SNPs were validated as with the IVS on chromosomes 1, 5, 6, and 7. On chromosome 2, the two QTLs detected in the full set were confirmed; meanwhile, 2/3 and 2/6 were validated on chromosomes 3 and 4, respectively, although three of them were not validated with the IVS (**Tables 2**, **3**). In summary, out of the 21 QTLs associated with tunnel length in the whole MAGIC population, 14 were validated. Nine were validated using both IVS and HVS, QTL_8_2 with IVS only, and QTL_2_2, QTL_3_3, QTL_4_2, and QTL_4_3 with HVS only (**Tables 2**, **3**).

**Table 2:** Validation of QTL found for tunnel length in the whole MAGIC population (Jiménez-Galindo et al., 2019) using a 56-inbred subset of the MAGIC population (MAGIC subset).

SNP^1^ (Jiménez-Galindo et al., 2019)	QTL^2^	*p-*value^3^	SNP^4^	*p-*value^5^	Distance (bp)^6^	LD *r* ^27^
Grain yield						
S1_3708430	QTL_1_1	0.01	S1_3708430	0.007	0	Same SNP
S1_199075640	QTL_1_2	0.95	S1_196201034	0.013	2,874,606	0.07
S1_199075673	QTL_1_2	0.95				
S1_199075674	QTL_1_2	0.95				
S1_199075675	QTL_1_2	0.95				
S1_199075677	QTL_1_2	0.95				
S1_199075679	QTL_1_2	0.95				
S1_199075681	QTL_1_2	0.95				
S1_199075682	QTL_1_2	0.95				
S1_199075684	QTL_1_2	0.95				
S1_200077059	QTL_1_3	0.35	S1_201741572	0.013	1,664,513	0.27
S1_200077088	QTL_1_3	0.35				
S1_200408507	QTL_1_3	0.54				
S1_200408528	QTL_1_3	0.47				
S1_200479419	QTL_1_3	0.57				
S1_200801543	QTL_1_3	0.27				
S1_201847366	QTL_1_4	0.32	S1_201741572	0.013	105,794	0.45
S1_201872641	QTL_1_4	0.52				
S1_202160398	QTL_1_4	0.44				
S1_202647745	QTL_1_4	0.49				
S1_279899012	QTL_1_5	0.76	S1_276323139	0.014	3,575,873	0.07
S1_298985014	QTL_1_6	0.63	S1_300073024	0.005	1,088,010	0.37
S1_298986682	QTL_1_6	0.99				
S3_200876919	QTL_3_1	0.22	S3_197019251	0.020	3,857,668	0.13
S3_200876939	QTL_3_1	0.22				
S3_223537342	QTL_3_2	0.19	S3_222132763	0.012	1,404,579	0.22
S3_229992268	QTL_3_3	0.63	S3_223745145	0.016	6,247,123	0.11
S4_235761612	QTL_4_1	0.47	S4_236393647	0.019	632,035	0.28
S5_128333604	QTL_5_1	0.58	S5_133776816	0.016	5,443,212	0.06
S5_128333610	QTL_5_1	0.58				
S7_33885476	QTL_7_1	0.10	S7_34480421	0.012	594,945	0.76
S8_171634738	QTL_8_1	0.32	S8_173704033	0.002	2,069,295	0.17
S9_5656122	QTL_9_1	0.21	S9_5656939	0.004	817	No
S9_5656138	QTL_9_1	0.21				
S9_9966270	QTL_9_2	0.08	S9_13495777	0.008	3,529,507	0.23
S9_9966272	QTL_9_2	0.08				
S9_9966291	QTL_9_2	0.08				
S9_11084679	QTL_9_3	0.09	S9_13495777	0.0080057	2,411,098	0.15
S9_11746822	QTL_9_4	0.52	S9_11564902	0.005	181,920	No

SNPs highlighted in green are validated following the two criteria previously described, i.e., the same SNP was associated with the trait or an SNP within an interval was associated with the trait.

^1^SNP identified by Jiménez-Galindo et al. (2019) as associated with grain yield. The number before the underscores indicates the chromosome number and the number after the underscore indicates the physical position in bp within the chromosome.

^2^QTL code from the whole MAGIC population (Jiménez-Galindo et al., 2019). The number before the underscores indicates the chromosome and the number after the underscores indicates the QTL within the chromosome.

^3^p-value of the significant SNP using the whole MAGIC (Jiménez-Galindo et al., 2019) in the GWAS of the MAGIC subset.

^4^Significant SNP at p < 0.02 in the GWAS of the MAGIC subset and located within the confidence interval of a significant SNP in the whole MAGIC (Jiménez-Galindo et al., 2019). The number before the underscores indicates the chromosome number and the number after the underscore indicates the physical position in bp within the chromosome.

^5^p-value of the significant SNP at p < 0.02 in the GWAS of the MAGIC subset.

^6^Distance in bp from the SNP described in Jiménez-Galindo et al. (2019) and the one found in the current study using the MAGIC subset.

^7^Linkage disequilibrium between the SNP described in Jiménez-Galindo et al. (2019) and the one in the current study using the MAGIC subset.

**Table 3:** Validation of significant QTL found for tunnel length in the whole MAGIC population (Jiménez-Galindo et al. 2019) in a hybrid background.

SNP^1^ (Jiménez-Galindo et al., 2019)	QTL^2^	*p-*value^3^	SNP^4^	*p-*value^5^	Distance (bp)^6^	LD *r* ^27^
Tunnel length						
S1_19252698	QTL_1_1	0.25	S1_18200092	0.002	1,052,606	0.22
S1_290934634	QTL_1_2	0.60	S1_290306138	0.004	628,496	0.01
S2_14798875	QTL_2_1	0.01	S2_14798875	0.009	0	Same SNP
S2_179803199	QTL_2_2	0.09	S2_180155965	0.015	352,766	0.77
S3_191332395	QTL_3_1	0.94	S3_190325618	0.018	1,006,777	0.45
S3_212770896	QTL_3_2	0.74	S3_212771115	0.004	219	0.15
S3_218807815	QTL_3_3	0.99	S3_218835769	0.018	27,954	0.31
S3_218807820	QTL_3_3	0.99				
S4_127856740	QTL_4_1	0.59	S4_131837902	0.014	3,981,162	0.11
S4_127955231	QTL_4_1	0.69				
S4_155128691	QTL_4_2	0.87	S4_154625109	0.003	503,582	0.21
S4_155830369	QTL_4_3	0.20	S4_154625109	0.003	1,205,260	0.39
S4_155830370	QTL_4_3	0.20				
S4_155830400	QTL_4_3	0.20				
S4_156193095	QTL_4_4	0.32	S4_156023370	0.006	169,725	0.02
S4_181340312	QTL_4_5	0.22	S4_201364747	0.016	20,024,435	0.21
S4_221752511	QTL_4_6	0.10	S4_222702242	0.019	949,731	No
S5_24771445	QTL_5_1	0.18	S5_33610351	0.016	8,838,906	0.38
S6_147725553	QTL_6_1	0.17	S6_147968131	0.011	242,578	1.00
S6_150800759	QTL_6_2	0.07	S6_150889722	0.011	88,963	0.68
S6_156035854	QTL_6_3	0.47	S6_156282464	0.017	246,610	0.69
S6_164776991	QTL_6_4	0.28	S6_160234735	0.013	4,542,256	0.33
S7_109722251	QTL_7_1	0.15	S7_112215633	0.005	2,493,382	0.42
S8_24527783	QTL_8_1	0.40	S8_26089118	0.016	1,561,335	0.01
S8_28525990	QTL_8_2	0.81	S8_26089118	0.016	2,436,872	0.03

SNPs highlighted in green are validated following the two criteria previously described, i.e., the same SNP was associated with the trait or an SNP within an interval was associated with the trait.

^1^SNP identified by Jiménez-Galindo et al. (2019) as associated with tunnel length. The number before the underscores indicates the chromosome number and the number after the underscore indicates the physical position in bp within the chromosome.

^2^QTL code from the whole MAGIC population (Jiménez-Galindo et al., 2019). The number before the underscores indicates the chromosome and the number after the underscores indicates the QTL within the chromosome.

^3^p-value of the significant SNP using the whole MAGIC (Jiménez-Galindo et al., 2019) in the GWAS of the MAGIC subset.

^4^Significant SNP at p < 0.02 in the GWAS of the MAGIC subset and located within the confidence interval of a significant SNP in the whole MAGIC (Jiménez-Galindo et al., 2019). The number before the underscores indicates the chromosome number and the number after the underscore indicates the physical position in bp within the chromosome.

^5^p-value of the significant SNP at p < 0.02 in the GWAS of the MAGIC subset.

^6^Distance in bp from the SNP described in Jiménez-Galindo et al. (2019) and the one found in the current study using the MAGIC subset.

Current GWAS was done on hybrids developed by crossing a 52-inbred subset of the MAGIC population (MAGIC subset) with A638.

**Table 4:** Validation of QTL found for grain yield in the whole MAGIC population (Jiménez-Galindo et al. 2019) using a 56-inbred subset of the MAGIC population (MAGIC subset).

SNP^1^ (Jiménez-Galindo et al., 2019)	QTL^2^	*p-*value^3^	SNP^4^	*p-*value^5^	Distance (bp)^6^	LD *r* ^27^
S1_3708430	QTL_1_1	0.007	S1_3197323	0.014	511,107	0.39
S1_199075640	QTL_1_2	0.953	S1_200288001	0.014	1,212,361	0.37
S1_199075673	QTL_1_2	0.953				
S1_199075674	QTL_1_2	0.953				
S1_199075675	QTL_1_2	0.953				
S1_199075677	QTL_1_2	0.953				
S1_199075679	QTL_1_2	0.953				
S1_199075681	QTL_1_2	0.953				
S1_199075682	QTL_1_2	0.953				
S1_199075684	QTL_1_2	0.953				
S1_200077059	QTL_1_3	0.353	S1_200288001	0.014	210,942	0.46
S1_200077088	QTL_1_3	0.353				
S1_200408507	QTL_1_3	0.545				
S1_200408528	QTL_1_3	0.471				
S1_200479419	QTL_1_3	0.572				
S1_200801543	QTL_1_3	0.270				
S1_201847366	QTL_1_4	0.319	S1_201872818	0.008	25,452	0.68
S1_201872641	QTL_1_4	0.521				
S1_202160398	QTL_1_4	0.440				
S1_202647745	QTL_1_4	0.490				
S1_279899012	QTL_1_5	0.762	S1_280826390	0.017	927,378	0.07
S1_298985014	QTL_1_6	0.628	S1_299470567	0.008	483,885	0.12
S1_298986682	QTL_1_6	0.985				
S3_200876919	QTL_3_1	0.220	S3_199293659	0.010	1,583,260	0.10
S3_200876939	QTL_3_1	0.220				
S3_223537342	QTL_3_2	0.192	S3_225038503	0.004	1,501,161	0.459
S3_229992268	QTL_3_3	0.634	S3_228983201	0.009	1,009,067	0.133
S4_235761612	QTL_4_1	0.466	S4_235381320	0.020	380,292	0.531
S5_128333604	QTL_5_1	0.583	S5_135433904	0.018	7,100,300	0.537
S5_128333610	QTL_5_1	0.583				
S7_33885476	QTL_7_1	0.101	S7_23960799	0.016	9,924,677	0.227
S8_171634738	QTL_8_1	0.323	S8_171625362	0.013	9,376	0.06
S9_5656122	QTL_9_1	0.207	S9_8039883	0.011	2,383,761	No
S9_5656138	QTL_9_1	0.207				
S9_9966270	QTL_9_2	0.082	S9_8039883	0.011	1,926,387	0.1
S9_9966272	QTL_9_2	0.082				
S9_9966291	QTL_9_2	0.082				
S9_11084679	QTL_9_3	0.086	S9_13662469	0.011	2,577,790	0.32
S9_11746822	QTL_9_4	0.525	S9_12599906	0.00696272	853,084	No

Current GWAS was done on hybrids developed by crossing a 52-inbred subset of the MAGIC population (MAGIC subset) with A638. SNP highlighted in green are validated following the two criteria previously described, i.e. the same SNP was associated with the trait or a SNP within an interval was associated with the trait ^1^SNP identified by Jiménez-Galindo et al. (2019) as associated with grain yield. The number before the underscores indicates the chromosome number and the number after the underscore indicates the physical position in bp within the chromosome. ^2^QTL code from the whole MAGIC population (Jiménez-Galindo et al. 2019). The number before the underscores indicates the chromosome and the number after the underscores indicates the QTL within the chromosome. ^3^p-value of the significant SNP using the whole MAGIC (Jiménez-Galindo et al. 2019) in the GWAS of the MAGIC subset ^4^Significant SNP at p < 0.02 in the GWAS of the MAGIC subset and located within the confidence interval of a significant SNP in the whole MAGIC (Jiménez- Galindo et al. 2019). The number before the underscores indicates the chromosome number and the number after the underscore indicates the physical position in bp within the chromosome. ^5^p-value of the significant SNP at p < 0.02 in the GWAS of the MAGIC subset ^6^Distance in bp from the SNP described in Jiménez-Galindo et al. 2019 and the one found in the current study using the MAGIC subset ^7^Linkage disequilibrium between the SNP described in Jiménez-Galindo et al. 2019 and the one in the current study using the MAGIC subset7: Linkage disequilibrium between the SNP described in Jiménez-Galindo et al. 2019 and the one in the current study using the MAGIC subset

### Validation of QTL for grain yield

**Figure 1:**
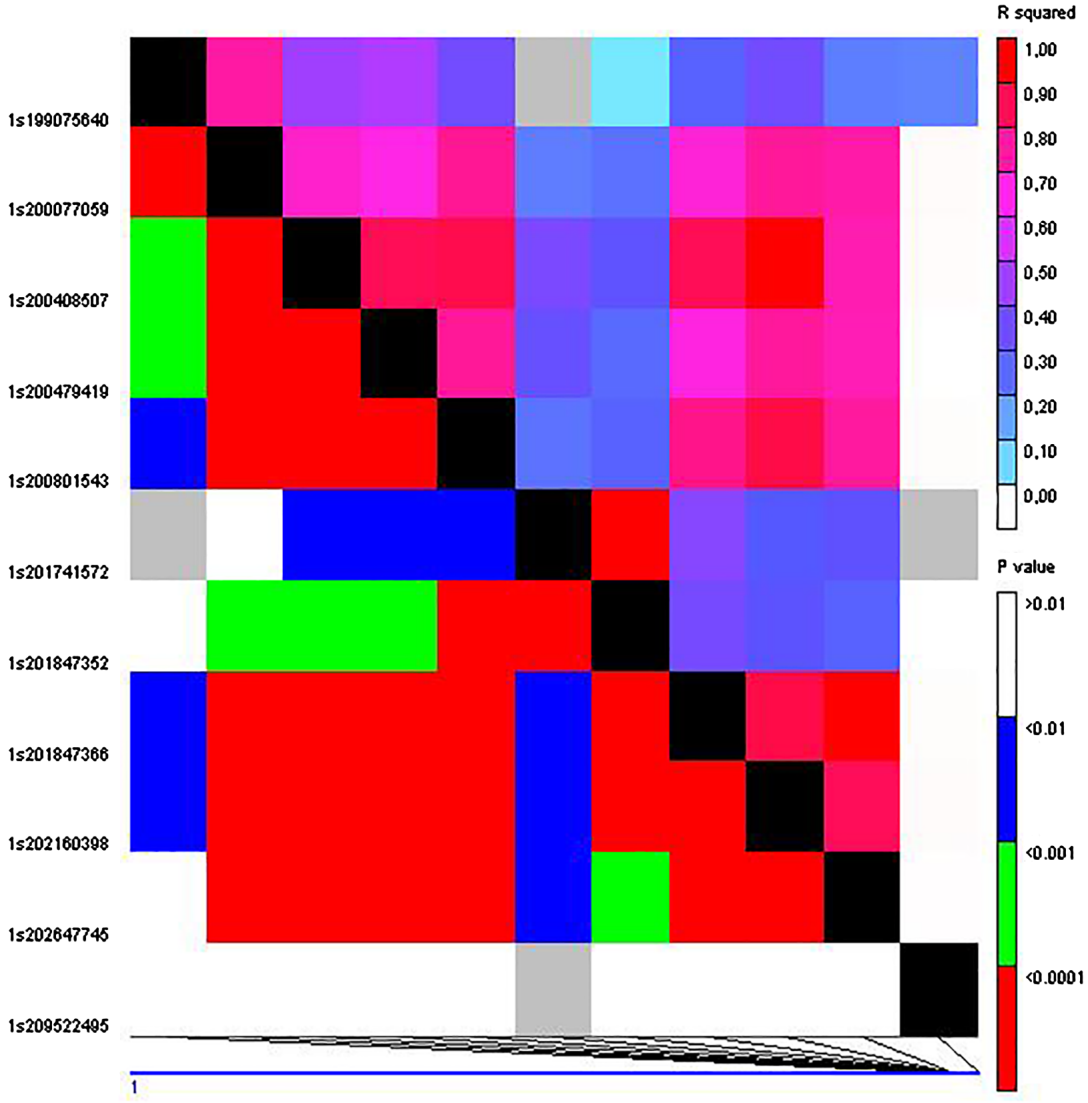
Linkage disequilibrium among the 23 SNPs on chromosome 1 significantly associated with grain yield using the whole MAGIC population (Jiménez-Galindo et al., 2019).

The SNP S1_3708430 that was described as significantly associated with grain yield in the complete set of RILs (Jiménez-Galindo et al., 2019) also showed a significant association (*p* < 0.02) with yield in the current study using the IVS. In addition, three out of the six QTLs for yield found on chromosome 1 using the complete MAGIC population (Jiménez-Galindo et al., 2019) were validated using both validation sets, IVS and HVS (QTL_1_1; QTL_1_3 and QTL_1_4). QTL_1_6 was only validated in the IVS and QTL_1_2 was only validated using the HVS (**Tables 4**, **5**). Notably, QTL_1_2 and QTL_1_3 exhibited a high degree of linkage disequilibrium (*r*2 = 0.80) and are situated at a distance of 1,001,419 base pairs (**Figure 1**). Similarly, the linkage between QTL_1_3 and QTL_1_4 is substantial (*r*2 = 0.72), with a genomic separation of 1,770,307 base pairs. This observation suggests the potential categorization of these adjacent QTLs as a single QTL encompassing the QTL_1_2, QTL_1_3, and QTL_1_4 reported by Jiménez-Galindo et al. (2019).

**Table 5:** Validation of significant QTL found for grain yield in the whole MAGIC population (MAGIC) and published in Jiménez-Galindo et al. (2019) in a hybrid background.

SNP^1^ (Jiménez-Galindo et al., 2019)	QTL^2^	*p-*value^3^	SNP^4^	*p-*value^4^	Distance (bp)^6^	LD r^27^
Tunnel length						
S1_19252698	QTL_1_1	0.12	S1_18978470	0.013	274,228	0.26
S1_290934634	QTL_1_2	0.13	S1_292415110	0.004	1,480,476	0.06
S2_14798875	QTL_2_1	0.15	S2_15140966	0.016	342,091	0.65
S2_179803199	QTL_2_2	0.68	S2_185171168	0.011	5,367,969	0.18
S3_191332395	QTL_3_1	0.16	S3_191368808	0.017	36,413	0.65
S3_212770896	QTL_3_2	0.86	S3_212502290	0.003	268,606	0.08
S3_218807815	QTL_3_3	0.66	S3_216256039	0.008	2,551,776	0.20
S3_218807820	QTL_3_3	0.66				
S4_127856740	QTL_4_1	0.35	S4_111050431	0.018	16,806,309	0.61
S4_127955231	QTL_4_1	0.38				
S4_155128691	QTL_4_2	0.94	S4_158606264	0.007	3,477,573	0.02
S4_155830369	QTL_4_3	0.37	S4_153520875	0.014	2,309,494	0.02
S4_155830370	QTL_4_3	0.37				
S4_155830400	QTL_4_3	0.37				
S4_156193095	QTL_4_4	0.27	S4_156718547	0.018	525,452	0.09
S4_181340312	QTL_4_5	0.86	S4_181696701	0.007	356,389	0.12
S4_221752511	QTL_4_6	0.73	S4_222716155	0.014	963,644	0.04
S5_24771445	QTL_5_1	0.09	S5_23764335	0.011	1,007,110	0.40
S6_147725553	QTL_6_1	0.93	S6_148774898	0.009	1,049,345	0.21
S6_150800759	QTL_6_2	0.15	S6_150697524	0.006	103,235	0.34
S6_156035854	QTL_6_3	0.14	S6_155739471	0.019	296,383	0.23
S6_164776991	QTL_6_4	0.85	S6_166815163	0.011	2,038,172	0.23
S7_109722251	QTL_7_1	0.87	S7_112219931	0.008	2,497,680	0.32
S8_24527783	QTL_8_1	0.96	S8_27642137	0.011	3,114,354	0.13
S8_28525990	QTL_8_2	0.66	S8_27642137	0.018	883,853	0.23

SNPs highlighted in green are validated following the two criteria previously described, i.e., the same SNP was associated with the trait or an SNP within an interval was associated with the trait.

^1^SNP identified by Jiménez-Galindo et al. (2019) as associated with tunnel length. The number before the underscores indicates the chromosome number and the number after the underscore indicates the physical position in bp within the chromosome.

^2^QTL code from the whole MAGIC population (Jiménez-Galindo et al., 2019). The number before the underscores indicates the chromosome and the number after the underscores indicates the QTL within the chromosome.

^3^p-value of the significant SNP using the whole MAGIC (Jiménez-Galindo et al., 2019) in the GWAS of the MAGIC subset.

^4^Significant SNP at p < 0.02 in the GWAS of the MAGIC subset and located within the confidence interval of a significant SNP in the whole MAGIC (Jiménez-Galindo et al., 2019).

^5^p-value of the significant SNP at p < 0.02 in the GWAS of the MAGIC subset.

^6^Distance in bp from the SNP described in Jiménez-Galindo et al. (2019) and the one found in the current study using the MAGIC subset.

^7^Linkage disequilibrium between the SNP described in Jiménez-Galindo et al. (2019) and the one in the current study using the MAGIC subset.

One out of the three QTLs for yield was found on chromosome 3 using the whole MAGIC, and the QTL on chromosome 7 and the QTL on chromosome 4 were validated using both validation sets. The QTL on chromosome 5 and one out of four QTLs on chromosome 9 were validated in HVS. Finally, one out of four QTLs on chromosome 9 was validated in the IVS. To summarize, out of the 39 SNPs, corresponding to 17 QTLs, associated with grain yield in the whole MAGIC population, 11 were validated. Six were validated using IVS and HVS, two with IVS only, and three with HVS only (**Tables 4**, **5**).

## Discussion

The experimental design used in Jiménez-Galindo et al., 2019 was an augmented design, originally conceived to evaluate a large number of genotypes with the objective of discarding those that presented poor performances. In this study, we have selected a reduced set of inbred lines (IVS) at the tails of distributions for resistance and yield in the whole MAGIC population in order to maintain trait variability but limiting the number of individuals. This allowed us to improve the phenotyping reliability as the new set could be evaluated using an α-lattice design with two replications instead of an augmented design. The α-lattice experimental design provides enhanced control over experimental variability among replications and blocks, improving phenotyping precision and repeatability and increasing the genotypic/phenotypic variation ratio. As a result of the improved experimental design and more accurate phenotyping, we observed a gain in heritability values in both validations sets, inbred lines and hybrids, compared to heritability estimates in the whole MAGIG population (Jiménez et al., 2019). The increased inheritance estimates for tunnel length and yield in the IVS seemed to be a consequence of higher observed genetic variability; meanwhile, observed genetic variance was not increased in the HVS but the higher number of evaluations reduced the variability of hybrid means. As expected, owing to the reduced importance of additive genetic effects in a heterozygous background, we found that hybrids presented half or less than a half of the genotypic variation of the *per se* inbreds agreeing with (Michel et al., 2022). The reduction of genetic variance in the HVS compared to IVS was significant only for tunnel length because additive effects are the only significant genetic effects for this trait and, consequently, dominance and/or epistasis effects would not contribute to increase genotypic variability among hybrids as it could happen for yield (Butrón et al., 1999, Cartea et al., 1999).

The QTL validation approach used in the current study greatly differed from approaches used in other works, as those studies usually restringed genetic variation to a particular genomic region (single QTL) using near-isogenic inbred lines (NIL) or heterogeneous inbred families (HIF) (Huo et al., 2016; Jiménez-Galindo et al., 2018; Zhao et al., 2022; Yang et al., 2017). Evaluation of NIL and HIF genotypes is very valuable to advance in the isolation and cloning of the genes involved in the inheritance of a particular trait, but is not useful to validate the genetic weight of a particular QTL in a more real scenario in which variation for the trait is not limited. Conversely, results derived from the current validations could provide information of the usefulness of QTL and genomic predictions, both based on the evaluation of the whole MAGIC population, for implementing marker-assisted or/and genomic selection. In addition, the validation of additive-effect QTL detected by Jiménez-Galindo et al. (2019) has been done in homozygous and heterozygous backgrounds, which would allow us to assess the value of these QTLs for improved hybrid development. For validation in a heterozygous background, IVS inbreds were crossed to the Reid inbred line A638. It has been previously shown that using testers from complementary heterotic groups to the heterotic group or groups of the evaluated inbreds is superior for detecting/validating additive-effect QTLs than testers more genetically related to the inbreds (Frascaroli et al., 2009).

In the present study, almost three-fourths of QTLs found as associated to tunnel length by Jiménez-Galindo et al. (2019) were validated. However, the *p-*values of current associations were high, excluding the suitability of these QTLs for performing MAS but emphasizing that genomic predictions based on genotype–phenotype associations in the whole MAGIC population could be valuable for genomic selection. QTLs in the region 6.05–6.07 were validated in both sets, and this genomic region has also been associated with resistance to stalk tunneling in different mapping populations and environments. In addition, this region encompasses several QTLs for cell wall digestibility (Ralph et al., 2004) and, consequently, deserves to be deeply studied to identify the genes behind those associations. Ten out of 16 (approximately ⅗) QTLs for grain yield found in the whole MAGIC population were validated. The QTL validation ratio was inferior for yield than for tunnel length as expected because dominance and/or epistatic effects are equally or more important than additive effects for yield and could mask additive effects (Butrón et al., 2009). Interestingly, the additive-effect QTL on the long arm of chromosome 8 detected by Jiménez-Galindo et al. (2019) could not be validated in any validation set and, in the same chromosome arm, several authors have found significant QTLs for mid-parent heterosis for yield (Schön et al., 2010.; Tang et al., 2010; Larièpe et al., 2012; Samayoa et al., 2017). Again, *p-*values of associations found in the current study between markers linked to QTLs detected by Jiménez-Galindo et al. (2019) and grain yield were high, suggesting that genomic prediction based on the evaluation of the whole MAGIC population could be suitable to improve yield. Gesteiro et al. (2023) have already shown that genotypic selection for yield surpassed phenotypic selection, although genotypic and phenotypic selection for reducing tunnel lengths could be preferred to improve yield and resistance simultaneously.

Nine out of 21 and 6 out of 17 QTLs identified for tunnel length and grain yield, respectively, by Jiménez-Galindo et al. (2019) were validated across both validation sets, suggesting a moderate genetic correlation between *per se* and testcross performance of inbred lines. However, Zhang et al. (2011), although having estimated moderate to high genetic correlations between *per se* and testcross evaluations for plant and ear heights, showed that few QTLs were detected in both genetic backgrounds. In any case, the validation of a moderate proportion of QTL for tunnel length in both validation sets suggested that genomic predictions for tunnel length based on inbred line evaluations could be useful to develop improved hybrids (Ma et al., 2022). Michel et al. (2022) already estimated promising genomic value predictive abilities between *r* = 0.49 and *r* = 0.55 for models predicting maize hybrid traits that were trained with *per se* data.

## Conclusions

In this study, we have used a novel validation QTL approach together with improved experimental designs that led to higher heritability estimates for both traits compared to the whole MAGIC population. A substantial proportion of QTLs associated with tunnel length and grain yield were validated across both sets. The current validations offer insights for guiding the implementation of marker-assisted or genomic selection strategies. Moreover, the study indicates the potential value of utilizing genomic predictions based on inbred line assessments to enhance the development of superior hybrid varieties.

Phenotyping genetically-wide panels of inbred lines or RILs is challenging due to the difficulty of controlling the high experimental error associated with large trials. Therefore, the high experimental errors associated with these evaluations could greatly interfere with QTL identification making necessary to validate those QTL before implementing any maker-assisted or genomic selection program. In such scenario, a novel validation approach has been used that allows to ascertain the reliability of QTL as well as its practical value to contribute to hybrid improvement.

## Data Availability

The original contributions presented in the study are included in the article/**Supplementary Material**. Further inquiries can be directed to the corresponding author.
