# Supplementary material for: Validation of QTLs associated with corn borer resistance and grain yield: implications in maize breeding
Source: Front Plant Sci. 2024 Oct 22;15:1404881. doi: 10.3389/fpls.2024.1404881 (PMC11536317; doi:10.3389/fpls.2024.1404881)
Supplement: Supplementary file 1 [file Table1.pdf]

Supplementary Table 1. BLUE estimates of RILs of the whole MAGIC population for tunnel length and yield per plant evaluated across teo years under Corn Borer infestation published in Jimenez-Galindo et al. 2019

RIL	Tunnel length (cm)	Yield per plant (g/plant)
EPS21LR_104	23.14	58.65
EPS21LR_109	19.69	35.68
EPS21LR_10	16.77	18.27
EPS21LR_110	15.32	30.91
EPS21LR_111	16.27	34.67
EPS21LR_112	24.95	73.86
EPS21LR_113	26.22	40.27
EPS21LR_116	26.08	40.03
EPS21LR_117	29.05	14.54
EPS21LR_119	20.11	29.48
EPS21LR_11	25.73	17.08
EPS21LR_120	9.25	26.49
EPS21LR_123	21.31	43.27
EPS21LR_124	26.93	20.04
EPS21LR_125	18.39	40.65
EPS21LR_126	21.96	40.99
EPS21LR_127	35.10	42.14
EPS21LR_128	36.63	57.19
EPS21LR_12	23.77	39.17
EPS21LR_132	30.25	34.87
EPS21LR_135	23.63	24.93
EPS21LR_136	19.52	31.20
EPS21LR_137	28.37	13.05
EPS21LR_138	23.03	42.49
EPS21LR_139	26.58	28.78
EPS21LR_13	50.40	51.80
EPS21LR_143	20.26	53.99
EPS21LR_145	16.48	40.57
EPS21LR_146	46.18	25.83
EPS21LR_147	29.06	34.92
EPS21LR_148	17.80	31.55
EPS21LR_149	22.37	34.10
EPS21LR_150	33.08	49.30
EPS21LR_152	29.43	54.48
EPS21LR_153	25.21	34.47
EPS21LR_154	20.01	34.64
EPS21LR_159	27.95	32.23
EPS21LR_162	22.89	32.77
EPS21LR_163	23.45	35.47
EPS21LR_165	17.64	24.30
EPS21LR_167	23.25	46.95
EPS21LR_172	17.33	21.86

EPS21LR_173	18.06	51.32
EPS21LR_175	25.01	29.17
EPS21LR_176	16.63	26.95
EPS21LR_178	27.91	37.66
EPS21LR_17	24.32	35.07
EPS21LR_180	26.91	34.49
EPS21LR_183	27.16	22.54
EPS21LR_184	24.36	17.82
EPS21LR_186	19.51	28.73
EPS21LR_189	15.64	30.37
EPS21LR_19	42.18	45.22
EPS21LR_1	12.77	44.94
EPS21LR_20	17.33	24.32
EPS21LR_210	45.14	41.76
EPS21LR_212	24.29	9.03
EPS21LR_214	28.51	57.80
EPS21LR_21	23.13	45.71
EPS21LR_239	30.03	63.66
EPS21LR_240	20.69	29.83
EPS21LR_241	25.72	36.12
EPS21LR_242	29.45	32.99
EPS21LR_243	28.60	55.98
EPS21LR_244	27.35	24.94
EPS21LR_245	25.77	20.03
EPS21LR_246	11.46	26.45
EPS21LR_247	31.32	44.43
EPS21LR_248	24.34	53.06
EPS21LR_249	21.20	44.45
EPS21LR_250	30.29	53.53
EPS21LR_251	27.31	38.13
EPS21LR_252		27.28
EPS21LR_253	11.50	41.13
EPS21LR_254	25.59	53.05
EPS21LR_255	27.95	50.50
EPS21LR_256	28.55	53.60
EPS21LR_257	36.84	45.92
EPS21LR_258	31.36	44.59
EPS21LR_259	25.37	79.46
EPS21LR_25	37.02	47.92
EPS21LR_260	31.09	43.86
EPS21LR_261	30.60	38.01
EPS21LR_262	42.58	71.37
EPS21LR_263	14.84	41.43
EPS21LR_264	23.80	48.95
EPS21LR_265	24.92	31.96
EPS21LR_267	15.73	44.65

EPS21LR_268	14.96	32.07
EPS21LR_269	13.13	28.79
EPS21LR_26	21.53	66.81
EPS21LR_270	21.52	49.92
EPS21LR_271	20.05	37.11
EPS21LR_272	24.87	77.15
EPS21LR_273	22.64	38.86
EPS21LR_274	44.24	16.00
EPS21LR_275	27.03	36.44
EPS21LR_276	15.50	70.07
EPS21LR_277	21.44	37.45
EPS21LR_278	26.27	32.89
EPS21LR_279	26.38	23.15
EPS21LR_27	19.43	19.82
EPS21LR_280	17.91	25.63
EPS21LR_281	19.28	44.04
EPS21LR_282	16.95	23.23
EPS21LR_283	25.48	40.01
EPS21LR_284	16.24	47.59
EPS21LR_285	30.57	48.97
EPS21LR_286	17.55	52.36
EPS21LR_287	23.79	43.74
EPS21LR_288	19.29	14.75
EPS21LR_289	49.49	58.23
EPS21LR_28	16.72	34.63
EPS21LR_290	29.48	34.49
EPS21LR_291	24.22	47.07
EPS21LR_292	26.29	70.20
EPS21LR_293	37.05	36.08
EPS21LR_294	34.78	55.81
EPS21LR_295	23.73	32.02
EPS21LR_297	34.49	34.16
EPS21LR_298	40.01	18.67
EPS21LR_299	22.69	40.85
EPS21LR_29	24.90	34.40
EPS21LR_2	9.77	34.42
EPS21LR_300	13.54	37.28
EPS21LR_301	26.51	67.63
EPS21LR_302	27.99	19.76
EPS21LR_303	10.31	29.12
EPS21LR_304	22.56	34.94
EPS21LR_305	31.36	49.35
EPS21LR_306	29.76	27.10
EPS21LR_307	31.91	53.05
EPS21LR_308	23.89	53.00
EPS21LR_309	23.11	37.50

EPS21LR_30	19.74	21.77
EPS21LR_310	25.91	33.60
EPS21LR_311	29.09	39.39
EPS21LR_312	30.97	66.24
EPS21LR_313	21.14	29.40
EPS21LR_314	25.54	34.04
EPS21LR_315	28.26	49.79
EPS21LR_316	42.08	42.44
EPS21LR_317	26.28	62.11
EPS21LR_318	30.81	67.34
EPS21LR_31	25.15	26.25
EPS21LR_320	12.09	34.55
EPS21LR_321	30.98	43.87
EPS21LR_322	24.78	32.17
EPS21LR_323	30.48	39.82
EPS21LR_324	14.98	30.30
EPS21LR_325	18.83	38.70
EPS21LR_326	10.62	47.12
EPS21LR_327	24.80	41.21
EPS21LR_328	36.21	59.84
EPS21LR_32	17.28	43.09
EPS21LR_331	27.05	41.30
EPS21LR_333	18.69	27.22
EPS21LR_334	19.80	51.41
EPS21LR_335	26.10	37.09
EPS21LR_336	29.47	36.18
EPS21LR_337	23.53	37.17
EPS21LR_338	22.07	46.53
EPS21LR_340	30.54	53.72
EPS21LR_341	13.14	28.73
EPS21LR_342	19.29	28.85
EPS21LR_343	21.51	26.11
EPS21LR_344	28.58	48.51
EPS21LR_345	19.66	38.31
EPS21LR_347	28.48	44.60
EPS21LR_348	38.67	23.18
EPS21LR_349	27.87	53.23
EPS21LR_350	28.34	35.17
EPS21LR_351	16.91	53.60
EPS21LR_352	29.74	37.73
EPS21LR_354	47.78	49.36
EPS21LR_356	22.30	52.80
EPS21LR_357	29.62	59.18
EPS21LR_358	17.68	41.01
EPS21LR_35	15.56	37.89
EPS21LR_360	14.62	40.29

EPS21LR_361	29.15	46.84
EPS21LR_362	43.27	46.89
EPS21LR_363	35.65	49.17
EPS21LR_364	36.09	45.39
EPS21LR_365	18.64	35.53
EPS21LR_366	16.47	21.15
EPS21LR_367	39.91	33.25
EPS21LR_368	27.74	49.70
EPS21LR_369	22.50	31.38
EPS21LR_36	16.39	32.73
EPS21LR_370	23.55	55.42
EPS21LR_371	28.76	24.47
EPS21LR_373	32.16	40.95
EPS21LR_374	27.48	18.58
EPS21LR_375	32.54	44.34
EPS21LR_377	18.46	46.02
EPS21LR_379	14.03	42.10
EPS21LR_37	16.37	68.23
EPS21LR_380	39.43	40.60
EPS21LR_381	22.31	38.23
EPS21LR_382	19.38	31.61
EPS21LR_383	12.84	52.79
EPS21LR_384	56.64	15.67
EPS21LR_385	25.06	67.82
EPS21LR_387	17.55	37.61
EPS21LR_388	22.39	51.59
EPS21LR_389	31.15	59.11
EPS21LR_38	18.57	28.19
EPS21LR_390	24.51	33.01
EPS21LR_391	32.44	40.71
EPS21LR_392	24.84	45.87
EPS21LR_393	19.64	33.78
EPS21LR_394	39.55	44.64
EPS21LR_395	13.01	42.67
EPS21LR_396	28.27	30.66
EPS21LR_397	23.23	39.27
EPS21LR_398	21.65	19.36
EPS21LR_39	28.43	42.30
EPS21LR_3	19.60	32.09
EPS21LR_400	41.24	45.86
EPS21LR_401	26.59	21.18
EPS21LR_402	26.49	41.34
EPS21LR_406	27.81	29.41
EPS21LR_407	12.45	34.20
EPS21LR_408	24.56	36.28
EPS21LR_410	33.42	20.47

EPS21LR_411	20.34	40.11
EPS21LR_412	28.67	41.33
EPS21LR_413	15.54	29.83
EPS21LR_414	22.73	50.20
EPS21LR_415	21.89	49.20
EPS21LR_416	19.67	38.15
EPS21LR_418	20.29	31.17
EPS21LR_420	29.46	40.83
EPS21LR_421	26.32	45.17
EPS21LR_422	21.48	35.99
EPS21LR_423	31.94	33.51
EPS21LR_424	17.97	64.37
EPS21LR_425	22.28	48.32
EPS21LR_426	10.69	23.80
EPS21LR_427	22.05	30.10
EPS21LR_428	20.65	39.15
EPS21LR_429	25.34	34.34
EPS21LR_42	10.89	30.63
EPS21LR_430	18.65	44.51
EPS21LR_431	33.80	42.05
EPS21LR_432	18.76	41.67
EPS21LR_433	19.82	43.10
EPS21LR_434		10.56
EPS21LR_435	22.04	40.61
EPS21LR_437	44.07	47.19
EPS21LR_438	39.83	41.23
EPS21LR_439	25.09	14.83
EPS21LR_440	37.66	26.93
EPS21LR_441	20.69	38.78
EPS21LR_442	22.17	38.24
EPS21LR_443	38.44	44.82
EPS21LR_444	32.77	57.32
EPS21LR_445	17.53	50.59
EPS21LR_446	17.94	30.50
EPS21LR_448	28.20	34.00
EPS21LR_449	17.33	40.66
EPS21LR_450	31.96	38.10
EPS21LR_451	24.96	31.75
EPS21LR_452	30.77	48.29
EPS21LR_454	18.14	25.57
EPS21LR_455	15.93	19.81
EPS21LR_456	27.79	39.83
EPS21LR_458	23.62	45.00
EPS21LR_459	20.83	35.97
EPS21LR_45	23.37	34.97
EPS21LR_460	25.38	24.56

EPS21LR_462	31.67	54.10
EPS21LR_464	28.22	29.44
EPS21LR_465	21.14	35.29
EPS21LR_466	14.58	51.78
EPS21LR_467	32.30	40.98
EPS21LR_468	17.96	57.23
EPS21LR_469	18.49	49.26
EPS21LR_46	14.40	21.75
EPS21LR_470	23.09	49.27
EPS21LR_471	9.74	37.79
EPS21LR_472	19.03	20.67
EPS21LR_473	32.85	31.27
EPS21LR_474	20.06	30.01
EPS21LR_475	24.46	50.28
EPS21LR_476	15.99	36.30
EPS21LR_477	13.14	23.78
EPS21LR_478	27.74	21.23
EPS21LR_479	18.08	19.43
EPS21LR_47	20.69	43.28
EPS21LR_480	18.95	24.68
EPS21LR_481	25.44	38.26
EPS21LR_482	14.95	31.02
EPS21LR_483	25.27	42.52
EPS21LR_484	17.61	30.09
EPS21LR_485	16.55	23.12
EPS21LR_486	18.26	40.59
EPS21LR_487	36.55	52.19
EPS21LR_488	21.73	32.36
EPS21LR_489	10.81	50.34
EPS21LR_490	11.14	47.33
EPS21LR_491	32.28	25.27
EPS21LR_492	29.91	31.88
EPS21LR_493	18.57	43.87
EPS21LR_494	17.29	32.07
EPS21LR_495	28.96	26.30
EPS21LR_496	23.23	36.63
EPS21LR_499	27.01	50.11
EPS21LR_49	17.96	29.64
EPS21LR_4	17.61	57.89
EPS21LR_500	27.36	53.61
EPS21LR_501	27.06	33.14
EPS21LR_502	21.53	38.04
EPS21LR_503	15.54	28.20
EPS21LR_504	21.44	28.97
EPS21LR_505	19.47	29.72
EPS21LR_506	21.96	22.56

EPS21LR_507	33.62	21.43
EPS21LR_508	26.27	27.57
EPS21LR_509	26.71	38.31
EPS21LR_510	17.67	38.42
EPS21LR_511	34.79	46.31
EPS21LR_513	35.61	50.26
EPS21LR_514	30.61	45.78
EPS21LR_515	20.47	42.09
EPS21LR_516	12.94	43.68
EPS21LR_519	4.99	24.04
EPS21LR_51	30.07	43.42
EPS21LR_520	47.11	35.40
EPS21LR_521	18.25	46.59
EPS21LR_522	30.49	34.01
EPS21LR_523	25.12	52.70
EPS21LR_524	24.87	21.55
EPS21LR_525	27.13	56.66
EPS21LR_526	42.82	48.40
EPS21LR_527	22.59	31.15
EPS21LR_528	30.84	41.54
EPS21LR_529	17.69	32.15
EPS21LR_530	39.96	36.52
EPS21LR_531	39.04	61.89
EPS21LR_532	29.51	50.44
EPS21LR_533	34.11	53.91
EPS21LR_535	20.02	28.62
EPS21LR_536	58.12	32.05
EPS21LR_537	23.78	73.88
EPS21LR_538	30.76	47.44
EPS21LR_540	26.67	50.11
EPS21LR_541	42.01	26.29
EPS21LR_542	28.40	39.16
EPS21LR_543	46.25	51.10
EPS21LR_544	28.40	43.63
EPS21LR_545	29.63	51.57
EPS21LR_546	26.36	20.28
EPS21LR_547	34.49	53.14
EPS21LR_548	48.61	48.00
EPS21LR_549	18.60	57.23
EPS21LR_550	24.39	34.41
EPS21LR_551	17.96	48.19
EPS21LR_552	21.38	36.56
EPS21LR_553	36.72	49.89
EPS21LR_554	22.09	16.26
EPS21LR_555	43.79	28.42
EPS21LR_556	33.40	58.11

EPS21LR_557	30.43	20.23
EPS21LR_558	26.34	28.71
EPS21LR_559	18.96	43.40
EPS21LR_55	18.12	19.36
EPS21LR_560	26.05	37.57
EPS21LR_561	27.60	79.65
EPS21LR_562	13.92	24.65
EPS21LR_563	17.28	42.03
EPS21LR_565	36.07	28.90
EPS21LR_566	33.98	39.61
EPS21LR_567	13.48	43.11
EPS21LR_568	20.66	46.34
EPS21LR_569	27.66	40.93
EPS21LR_56	28.07	13.20
EPS21LR_570	23.50	67.02
EPS21LR_571	19.31	40.15
EPS21LR_572	17.64	35.36
EPS21LR_573	17.94	70.10
EPS21LR_574	21.83	44.80
EPS21LR_575	24.51	68.19
EPS21LR_576	21.38	41.44
EPS21LR_577	35.15	47.28
EPS21LR_578	27.07	36.78
EPS21LR_579	9.09	54.55
EPS21LR_580	26.40	34.41
EPS21LR_581	23.50	35.27
EPS21LR_582	30.53	41.15
EPS21LR_584	9.82	14.48
EPS21LR_585	28.05	70.18
EPS21LR_586	28.56	10.27
EPS21LR_587	30.58	26.77
EPS21LR_588	24.28	23.91
EPS21LR_589	36.43	43.62
EPS21LR_58	19.75	34.84
EPS21LR_590	29.81	40.62
EPS21LR_591	21.12	27.68
EPS21LR_592	29.10	48.16
EPS21LR_593	44.23	72.97
EPS21LR_594	23.52	52.04
EPS21LR_595	19.26	20.49
EPS21LR_596	26.12	35.06
EPS21LR_597	15.99	44.79
EPS21LR_598	22.61	47.72
EPS21LR_59	26.42	27.50
EPS21LR_5	16.83	34.12
EPS21LR_600	19.23	28.73

EPS21LR_601	34.73	52.35
EPS21LR_602	20.63	45.15
EPS21LR_603	15.21	49.30
EPS21LR_604	21.61	48.84
EPS21LR_605	29.70	50.27
EPS21LR_606	25.36	53.23
EPS21LR_607	20.97	47.05
EPS21LR_608	26.38	72.24
EPS21LR_60	29.17	40.72
EPS21LR_610	20.95	38.08
EPS21LR_611	29.99	43.96
EPS21LR_612	19.08	26.70
EPS21LR_613	29.02	31.78
EPS21LR_614	39.68	54.13
EPS21LR_616	32.92	38.65
EPS21LR_617	31.87	44.06
EPS21LR_618	19.40	23.34
EPS21LR_619	21.50	34.54
EPS21LR_620	33.98	39.11
EPS21LR_621	35.32	12.94
EPS21LR_622	25.90	6.59
EPS21LR_623	16.11	31.95
EPS21LR_624	23.25	39.97
EPS21LR_625	27.06	46.41
EPS21LR_626	8.09	25.83
EPS21LR_627	25.01	
EPS21LR_628	31.01	35.60
EPS21LR_630	34.97	42.81
EPS21LR_631	23.49	43.77
EPS21LR_632	20.90	28.52
EPS21LR_633	5.82	25.36
EPS21LR_634	20.40	33.75
EPS21LR_635	33.63	45.44
EPS21LR_636	38.17	46.18
EPS21LR_637	29.52	48.60
EPS21LR_638	17.32	41.08
EPS21LR_639	30.39	31.75
EPS21LR_63	27.54	24.65
EPS21LR_640	30.00	41.90
EPS21LR_641	25.44	56.28
EPS21LR_642	15.23	39.62
EPS21LR_643	25.87	61.28
EPS21LR_644	35.88	52.70
EPS21LR_645	32.10	35.78
EPS21LR_646	24.57	34.00
EPS21LR_647	20.55	63.95

EPS21LR_648	17.57	43.31
EPS21LR_649	27.09	36.72
EPS21LR_64	34.32	37.98
EPS21LR_652	30.50	40.81
EPS21LR_655	25.66	41.77
EPS21LR_657	35.62	58.12
EPS21LR_658	42.77	29.30
EPS21LR_659	31.16	33.52
EPS21LR_65	26.45	41.36
EPS21LR_660	34.75	46.97
EPS21LR_663	16.12	18.18
EPS21LR_664	39.49	33.70
EPS21LR_665	29.29	20.29
EPS21LR_666	46.80	25.77
EPS21LR_667	16.26	30.37
EPS21LR_668	28.45	42.37
EPS21LR_669	21.30	57.93
EPS21LR_66	16.76	43.42
EPS21LR_670	34.92	77.84
EPS21LR_673	24.04	41.99
EPS21LR_674	43.24	51.37
EPS21LR_675	24.58	39.46
EPS21LR_676	17.27	41.35
EPS21LR_677	20.61	70.67
EPS21LR_678	30.23	56.97
EPS21LR_679	19.44	58.04
EPS21LR_67	32.82	19.44
EPS21LR_680	23.79	36.35
EPS21LR_681	27.41	36.43
EPS21LR_682	28.44	49.47
EPS21LR_683	11.47	28.09
EPS21LR_684	43.06	38.83
EPS21LR_685	36.94	42.65
EPS21LR_686	35.31	69.25
EPS21LR_687	31.54	28.93
EPS21LR_688	27.14	31.63
EPS21LR_689	28.39	57.87
EPS21LR_68	18.90	33.57
EPS21LR_690	31.24	48.60
EPS21LR_691	29.27	56.07
EPS21LR_692	16.24	35.61
EPS21LR_693	19.84	26.26
EPS21LR_694	31.27	57.40
EPS21LR_695	16.38	22.74
EPS21LR_696	32.22	22.40
EPS21LR_697	26.54	30.87

EPS21LR_698	8.92	20.60
EPS21LR_69	23.13	26.30
EPS21LR_700	27.11	39.93
EPS21LR_701	31.74	36.16
EPS21LR_702	22.84	40.96
EPS21LR_703	31.47	20.95
EPS21LR_704	28.70	42.55
EPS21LR_705	41.71	34.76
EPS21LR_706	31.82	40.99
EPS21LR_707	27.36	49.70
EPS21LR_708	30.90	39.17
EPS21LR_709	23.59	25.99
EPS21LR_70	28.67	35.82
EPS21LR_710	30.77	38.15
EPS21LR_711	30.44	50.55
EPS21LR_712	33.63	39.43
EPS21LR_713	31.18	37.39
EPS21LR_714	31.88	29.92
EPS21LR_715	34.16	32.44
EPS21LR_716	25.30	35.54
EPS21LR_717	18.64	55.25
EPS21LR_718	12.46	39.08
EPS21LR_719	25.08	34.14
EPS21LR_71	43.42	24.07
EPS21LR_720	14.30	62.28
EPS21LR_721	13.91	34.35
EPS21LR_722	26.07	23.21
EPS21LR_723	15.91	34.31
EPS21LR_724	37.38	57.41
EPS21LR_725	27.97	83.44
EPS21LR_726	15.11	40.95
EPS21LR_727	17.87	59.88
EPS21LR_728	31.61	31.63
EPS21LR_729	41.95	39.69
EPS21LR_72	34.30	39.09
EPS21LR_730	24.02	7.83
EPS21LR_731	37.03	35.29
EPS21LR_732	27.54	48.60
EPS21LR_733	39.33	27.42
EPS21LR_734	17.27	50.47
EPS21LR_735	22.49	41.08
EPS21LR_736	26.07	31.43
EPS21LR_737	39.22	56.13
EPS21LR_738	37.65	48.41
EPS21LR_739	21.52	23.19
EPS21LR_740	50.46	71.43

EPS21LR_741	26.84	44.08
EPS21LR_742	30.43	52.51
EPS21LR_743	32.67	41.69
EPS21LR_745	22.36	58.11
EPS21LR_746	26.76	39.44
EPS21LR_747	37.20	58.13
EPS21LR_748	39.89	50.33
EPS21LR_749	33.69	36.81
EPS21LR_74	45.49	37.69
EPS21LR_750	26.02	43.85
EPS21LR_751	22.09	30.89
EPS21LR_752	25.64	45.10
EPS21LR_754	41.97	25.91
EPS21LR_755	23.09	31.15
EPS21LR_756	27.10	50.96
EPS21LR_757	26.11	20.73
EPS21LR_759	32.73	24.07
EPS21LR_75	20.05	36.73
EPS21LR_760	29.31	27.30
EPS21LR_761	22.32	25.65
EPS21LR_762	28.93	46.28
EPS21LR_763	25.61	51.96
EPS21LR_764	19.02	56.56
EPS21LR_766	28.56	19.54
EPS21LR_767	26.97	27.60
EPS21LR_768	27.06	50.23
EPS21LR_769	19.12	51.95
EPS21LR_770	39.81	62.26
EPS21LR_771	17.93	67.83
EPS21LR_772	17.63	26.04
EPS21LR_773	19.41	24.36
EPS21LR_774	17.61	1.54
EPS21LR_775	23.75	47.57
EPS21LR_777	31.01	14.60
EPS21LR_778	23.55	25.40
EPS21LR_779	30.41	32.50
EPS21LR_780	15.87	45.04
EPS21LR_781	21.80	40.60
EPS21LR_783	24.48	14.64
EPS21LR_784	26.56	30.27
EPS21LR_787	21.80	7.74
EPS21LR_788	19.51	50.21
EPS21LR_789	37.91	57.03
EPS21LR_78	23.49	3.04
EPS21LR_793	40.26	50.30
EPS21LR_795	31.76	21.50

EPS21LR_7	12.90	43.53
EPS21LR_80	38.38	45.98
EPS21LR_81	20.98	22.54
EPS21LR_82	10.99	44.94
EPS21LR_84	22.40	56.46
EPS21LR_85	16.17	20.82
EPS21LR_87	16.49	20.07
EPS21LR_88	19.50	26.38
EPS21LR_8	21.57	43.92
EPS21LR_95	29.26	38.60
EPS21LR_97	33.02	37.06
EPS21LR_99	15.20	47.46
EPS21LR_9	20.38	42.93
